# Dual attention network for unsupervised medical image registration based on VoxelMorph

**DOI:** 10.1038/s41598-022-20589-7

**Published:** 2022-09-28

**Authors:** Yong-xin Li, Hui Tang, Wei Wang, Xiu-feng Zhang, Hang Qu

**Affiliations:** 1 College of Mechanical and Electronic Engineering, Dalian Minzu University, Dalian, China; 2 Department of Health Sciences and Kinesiology, Georgia Southern University, Statesboro, 30458 USA; 3 Department of Radiology, Affiliated Hospital of Yangzhou University, Yangzhou, China

**Keywords:** Magnetic resonance imaging, Computational neuroscience, Computer science

## Abstract

An accurate medical image registration is crucial in a variety of neuroscience and clinical studies. In this paper, we proposed a new unsupervised learning network, DAVoxelMorph to improve the accuracy of 3D deformable medical image registration. Based on the VoxelMorph model, our network presented two modifications, one is adding a dual attention architecture, specifically, we model semantic correlation on spatial and coordinate dimensions respectively, and the location attention module selectively aggregates the features of each location by weighting the features of all locations. The coordinate attention module further puts the location information into the channel attention. The other is introducing the bending penalty as regularization in the loss function to penalize the bending in the deformation field. Experimental results show that DAVoxelMorph achieved better registration performance including average Dice scores (0.714) and percentage of locations with non-positive Jacobian (0.345) compare with VoxelMorph (0.703, 0.355), CycleMorph (0.705, 0.133), ANTs SyN (0.707, 0.137) and NiftyReg (0.694, 0.549). Our model increases both model sensitivity and registration accuracy.

## Introduction

Deformable image registration is crucial in a variety of clinical studies and applications since it aligns the image space into a common anatomical space. As the key technology of Image-aided diagnosis and treatment, registration technology can improve the efficiency of detecting the treatment effect. Meanwhile, this technology can maximize the fusion of medical images of different modes or times, and improve the utilization of information and the accuracy of diagnosis. Traditional registration methods attempt to estimate smooth deformation fields by optimizing cost functions associated with similarity metrics. However, these methods usually involve numerical optimization in high dimensions and are usually computationally expensive^1^. The emergence of deep learning-based methods has shown successfully addressed the limitations of conventional methods. A previous study pointed out that an unsupervised end-to-end learning strategy achieved a 100 × speed-up for 2D tissue registration compared to traditional image registration methods^2^. Fan et al. evaluated their BIRNet model in the 3D brain images. Compared to other deformable registration methods, their dual-guided fully convolutional neural network, BIRNet requires no iterative optimization and takes the least computational time^3^. In addition, registration accuracy has shown improved greatly in deep learning-based methods. For example, Cao et al. proposed a CNN-based regression model to directly learn the complex mapping from the input image pair to their corresponding deformation field. The evaluation of this model showed a maximal 2.6% improved dice similarity coefficient (DSC) in the white matter, gray Matter and cerebrospinal fluid registration^4^.

Recently the unsupervised registration framework became prominent due to the high challenging to obtain the real transformation and segmentation labels required by the supervised methods. de Vos et al. proposed the first unsupervised registration network DIRNet based on image similarity, taking the similarity between image pairs to be registered as a loss function, making end-to-end network training possible^5^. Yoo et al. and Sheikhjafari et al. used convolution self-encoder to encode the input image pairs to be registered into feature vectors and calculate the feature-based similarity loss^6,7^. The results showed that the feature-based similarity measurement method is better than the gray-scale similarity measurement method. Meanwhile, transformer has become very popular in a wide range of computer vision tasks. Vit and its derived instances have achieved the most advanced performance on multiple benchmark datasets^8^, showing the great potential in medical image analysis tasks. However, compared with convolutional network, transformer local information modeling lacks spatial induced bias, and the size of medical image data set is usually small, which makes it more difficult to learn the position coding of images^9–11^. Among all recent proposed unsupervised learning models, VoxelMorph model combines CNN and spatial transformation (ST) exceeded others^12^. It showed higher computational efficiency since it does not require a large amount of labeling data. It is a probabilistic generative model that defines registration as a parametric function and models functions using CNN and reconstruct images using ST layer. It is an inference algorithm based on unsupervised learning to provide diffeomorphic guarantees and uncertainty estimates, which learned the parameters by optimizing the variational lower bound^13–15^. However, we note that VoxelMorph fail to effectively suppress useless information on the spatial field during modeling the registration parameter function. Hence, in order to improve sensitivity issue of VoxelMorph, we propose a modified unsupervised learning model by employing the attention mechanism in the registration field to focus on important features and suppress unnecessary ones.

Our novel unsupervised learning model DAVoxelMorph for deformable medical image registration have two contributions. First, our CNN architecture module that combines the dual attention mechanism of coordinate attention and spatial attention. The module is superior in automatically learning different shapes and sizes of the target structure, implicitly learning to suppress irrelevant regions in the image during model training, and highlighting significant useful features for the registration task. Secondly, we introduce a modified loss function, which consists of cross-correlation and bending penalty regularization method. Our loss function has superior robustness and less susceptible to the effects of image grayscale distribution and contrast.

## Related work

### Deep learning-based registration

Depending on the type of annotation available in the training data, deep learning registration approaches can be broadly categorized as supervised, weakly supervised, and unsupervised transformation estimation^16^. The supervised learning requires the training datasets to include ground-truth deformation field, which is obtained either by simulating deformations and deformed images, or by running classical registration methods on pairs of scans. Based on a patch-based training system, Yang et al. design a deep encoder–decoder network to initialize the momentum of the large deformation diffeomorphic metric mapping registration model^17^. Sokooti et al. trained a 3D CNN to register chest CT data using artificially generated displacement vector field^18^. On the other hand, image-to-image prediction can be performed by a fully convolutional neural network (FCN), in which pixel-level features are predicted. For example, Fischer et al. proposed a novel CNN model for optical flow prediction, which trained end-to-end on a synthetic dataset and performed image-to-image optical flow prediction^19^.

Alternatively, some works have focused on the weakly supervised learning. For example, Hu et al.^20^, Xu and Niethammer^21^ showed networks trained to maximize the alignment between tissue labels. Besides, Blendowski et al. used a shape encoder–decoder network to extract cardiac shape representations as a basis for registration^22^. Drawbacks included the time-consuming nature of tissue labeling and the dependence on the performance of the resulting network on the accuracy of tissue labeling are well recognized^23^.

Although supervised methods have presented a promising direction, ground truth warp fields derived by traditional registration tools as ground truth can be laborious to acquire and can restrict the learned deformation types. In contrast, unsupervised learning mainly uses spatial transformer networks (STN) to warp moving image with estimated registration field, and training of the estimators relies on the design of data similarity function and smoothness of estimated registration field^24^. First unsupervised learning methods include Deep Learning Image Registration (DLIR) proposed by de Vos et al.^5^ and non-rigid image registration using FCN introduced by Li and Fan^25^. In addition, the starting point of the present work is the VoxelMorph framework. It is considered as the state-of-the-art, as is fully unsupervised and allows for a clinically feasible real-time solution by registering full 3D volumes in a single shot^12^. Given the large dataset the VoxelMorph model has been evaluated, it exceeded other medical registration methods. With an appropriate loss function such as mutual information, the model can perform multimodal registration.

### Attention mechanism

Attention mechanisms tells a model “what” and “where” to attend and have been proven helpful in a variety of computer vision tasks^26^, such as image classification^27,28^, and image segmentation^29,30^. Wang et al. introduced an encoder–decoder style attention module^31^. This high-capacity unit is inserted into deep residual networks between intermediate stages. In contrast, Hu et al. proposed the SE block, which is a lightweight gating mechanism. It specialized to model channel-wise relationships in a computationally efficient manner and designed to enhance the representational power of basic modules throughout the network^28^. However, the SE attention neglects the importance of positional information, which is critical to capturing object structures in vision tasks^32^. To exploit positional information, later works included BAM and CBAM attempt to reduce the channel dimension of the input tensor and then compute spatial attention using convolutions^33,34^. Given that convolutions can only capture local relations but fail to model long-range dependencies that are essential for vision tasks, Hou et al. proposed an efficient attention mechanism coordinate attention by embedding positional information into channel attention to enable mobile networks to attend overlarge regions while avoiding incurring significant computation overhead^35^. The coordinate attention block is another starting point for the present work. In our AttentionVoxelMoprh network, we introduce Dual Attention CNN Architecture by combining coordinate attention block and spatial attention block to further strengthen salient features and suppress useless information in the registration field.

### Loss function of image registration model

The loss function of a non-rigid image registration model based on unsupervised learning is usually composed of two parts. One part is the similarity measure between the reference image and the deformed floating image. The other is the spatial regularization of the deformation field predicted by the network to constrain the spatial smoothness of the deformation field. There are three commonly used image similarity measures: mean squared voxel difference, cross-correlation and mutual information. Mean Squared Voxel Difference and cross-correlation are usually used for unimodal images. The mutual information is usually used for multimodal images, which has better robustness in unimodal images. In the processing of network training, discontinuous deformation fields are often generated in the network in order to measure the similarity of images to the maximum extent. Therefore, it is usually necessary to apply spatial smoothing constraints on the predicted deformation field, that is, to penalize the spatial gradient of the deformation field, such as spatial regularization in VoxelMorph to calculate the square of the L2 norm of the gradient of the deformation field. Recent work has proposed a regularization method called bending penalty^36^, which computes the second-order gradient of the deformation field to penalize folding in the deformation field, and we will incorporate this into our loss function.

## Methods

On the basis of VoxelMorph framework, we propose an VoxelMorph Dual Attention CNN Architecture, an attention enhanced approach that further inhibit the useless information in the spatial field and improve the model accuracy. We learn the network parameters in an unsupervised fashion. We combine the attention modules that generate inter-spatial relationship, consider both positional information and channel-wise relationships. We confirm that all methods were carried out in accordance with relevant guidelines and this study was approved by the Ethics Committee of the Affiliated Hospital of Yangzhou University (2017-YKL11-021).

### VoxelMorph CNN architecture

**Figure 1:**
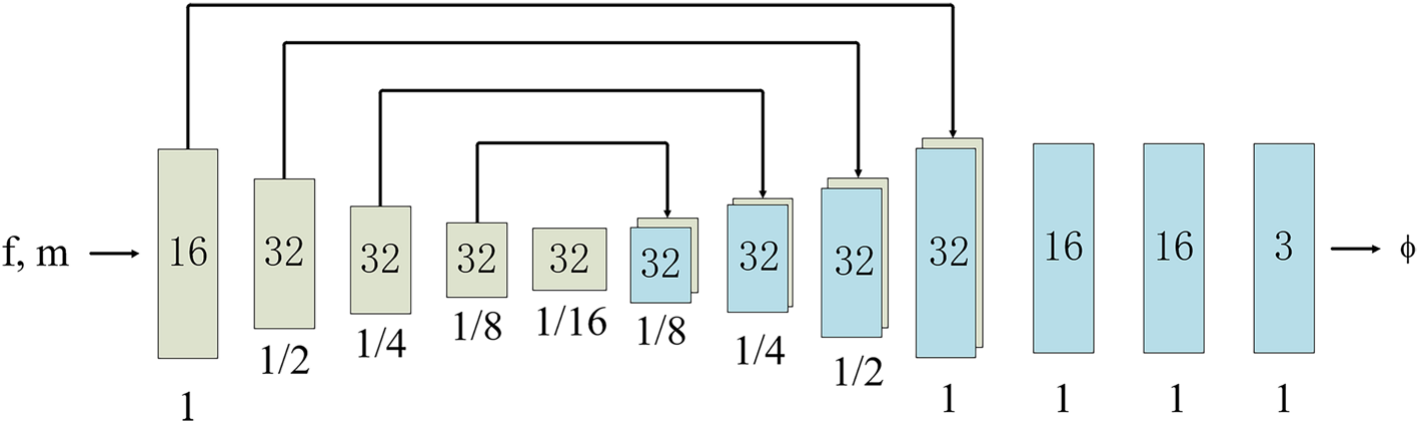
CNN architecture implemented gθ (f, m). Each rectangle represents a 3D volume, generated from the preceding volume using a 3D convolutional network layer. The spatial resolution of each volume with respect to the input volume is printed underneath.

VoxelMorph is an unsupervised registration framework based on convolutional neural network (CNN). It estimates the dense deformation field in one step by cascading U-Net and STN structures to realize deformable registration of 3D brain MRI images. Under unsupervised conditions, the registration accuracy can be significantly better than SyN^37^, which confirmed the superiority of unsupervised registration method over supervised and traditional registration methods. We use the same VoxelMorph CNN architecture proposed by Balakrishnan et al.^12,38^. The parameterization of *g*_*θ*_ (·,·) is based on a CNN convolutional neural network architecture that consists of encoder and decoder sections with skip connections. The VoxelMorph CNN architecture concatenates the moving image M and fixed image F into a 2-channel 3D image as the input and generate the corresponding ϕ (Fig. 1). In the encoder stage, 3 × 3 × 3 convolutions with stride 2 followed by Leaky ReLU activations are used to reduce the spatial dimensions until the bottleneck layer. In the decoder stage, we alternate between upsampling, convolutions and concatenating skip connections that propagate features learned during the encoding stages directly to layers generating the registration.

### Dual attention CNN architecture

In the medical images, attention needs to be focused on salient features (relevant tissues or organs) that are useful for a specific task, suppressing irrelevant regions in the input image. In cascaded neural networks, an explicit external tissue/organ localization module is required, and the use of spatial attention to learn individual elements with respect to the target can replace this operation. In order to make the most use of the spatial information extracted from the encoding and corresponding decoding stages, we propose a CNN architecture module that integrates the dual attention mechanism of coordinate attention and spatial attention.

#### Coordinate attention

**Figure 2:**
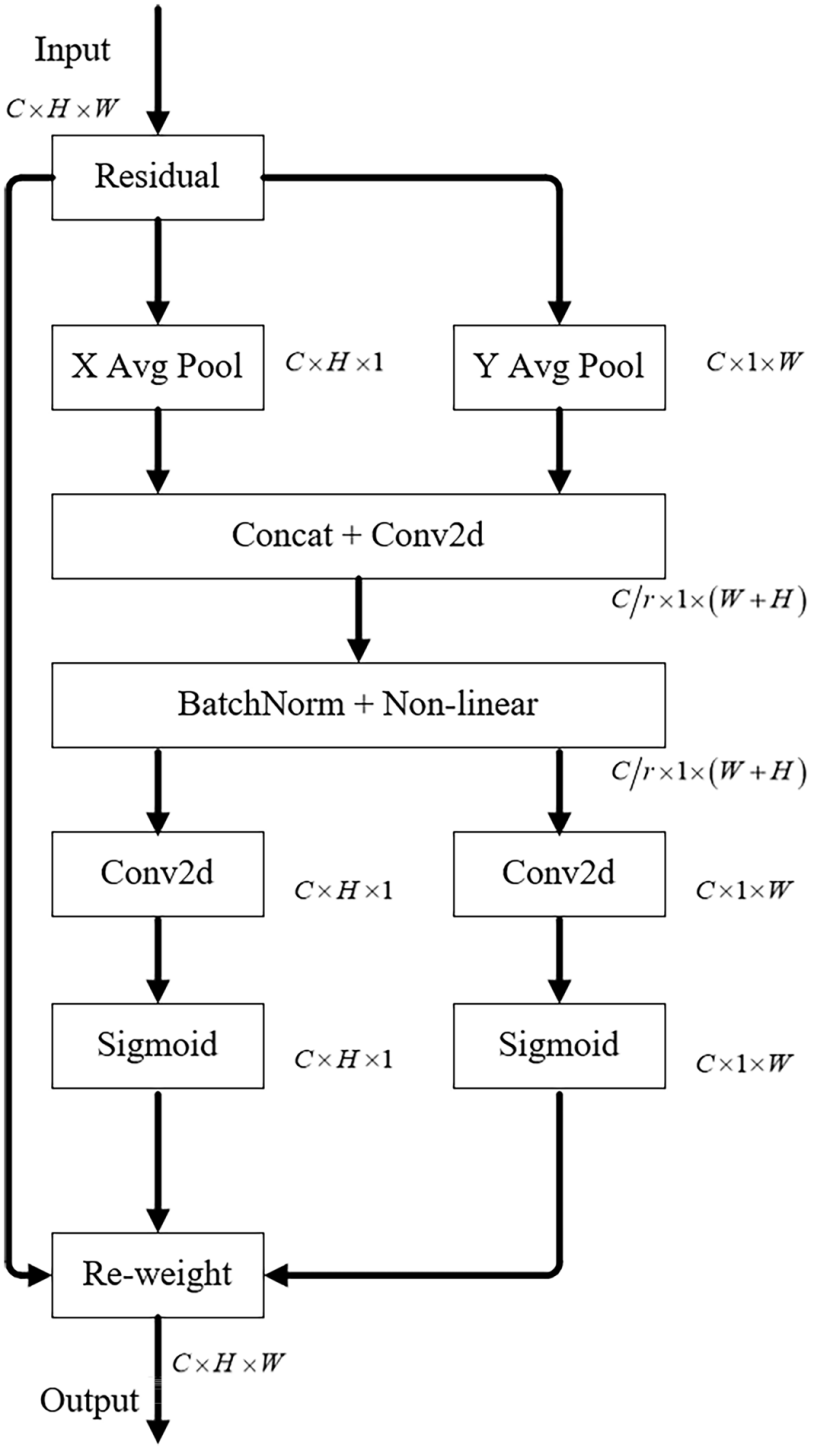
The coordinate attention block, where X Avg Pool and Y Avg Pool represent 1D horizontal global pooling and vertical global pooling, respectively.

Note that the standard convolution itself is difficult to model the channel relationships. However, to aggregate global information, global average pooling has been commonly adopted. In order to get channel-wise statistics, we first apply Squeeze- and- Excitation (SE) blocks proposed by^28^. Given the input X, the squeeze step for the c-th channel is calculated as follows:

1 $$ z_{c} = \frac{1}{H \times W}\sum\limits_{i = 1}^{H} {\sum\limits_{j = 1}^{W} {x_{c} } } (i,\;j) $$

where $z_{c}$ is the output related to the c-th channel. The input comes directly from a convolutional layer with a fixed kernel size and then be considered as a collection of local descriptors. Noticeably, the global pooling operation squeezes global spatial information into channel descriptors, causing the difficulty in preserving positional information. Therefore, we propose to introduce the coordinate attention blocks, demonstrating in Fig. 2. It is demonstrated by Hou et al.^35^, which considers both inter-channel relationships and positional information.

Two 1D global pooling operations are used to aggregate the input features into two separate directional perceptual feature maps along the vertical and horizontal directions respectively. Each attention map captures the long-distance dependence of the input feature map along one spatial direction. As shown in Fig. 2, the given input consists of two spatial extents of pooling kernels (H, 1) or (1, W) to encode each channel along the horizontal coordinate (X) and the vertical coordinate (Y), respectively. The output of c-th channel at height h can be formulated as Eq. (2), and the output of the vertical coordinate can be formulated as Eq. (3):

2 $$ z_{c}^{h} (h) = \frac{1}{W}\sum\limits_{0 \le i < W} {x_{c} } (h,\;i). $$

3 $$ z_{c}^{w} (w) = \frac{1}{H}\sum\limits_{0 \le j < H} {x_{c} } (j,\;w) $$

The two transformations described above aggregate characteristics along the two spatial directions, resulting in a pair of direction-aware feature maps. Demonstrated in the Fig. 2, X Aveg Pooling and Y Avg Pooling represents that the two transformations that enabling our attention block to capture long-range dependencies along one spatial dimension while preserving exact positional information along the other, which allow networks to detect objects of interest accurately.

After the coordinate information embedding stage mentioned above, we perform the coordinate attention generation by concatenating the aforementioned two transformations first and then use $1 \times 1$ convolutional transformation function *F*_1_:

4 $$ f = \delta \left( {F_{1} \left( {\left[ {z^{h} ,\;z^{w} } \right]} \right)} \right) $$

where $\left[ { \cdot, \cdot } \right]$ is the operation of concatenating along the spatial dimension, $\delta$ is the non-linear activation function, and ${\mathbf{f}} \in {\mathbb{R}}^{C/r \times (H + W)}$ is the intermediate feature map that encodes spatial information in both the horizontal and the vertical directions. *r* is the reduction ratio for controlling SE block. Then, splitting f along the spatial dimension into two separate tensors: ${\mathbf{f}}^{h} \in {\mathbb{R}}^{C/r \times H}$ and ${\mathbf{f}}^{w} \in {\mathbb{R}}^{C/r \times W}$. Apply another two 1 × 1 convolutional transformations $F_{h}$ and $F_{w}$ to transform ${\mathbf{f}}_{h}$ and ${\mathbf{f}}_{w}$ into tensors with same channel numbers to the input X. Equations (5) and (6) show as below:

5 $$ {\mathbf{g}}^{h} = \sigma \left( {F_{h} \left( {{\mathbf{f}}^{h} } \right)} \right) $$

6 $$ {\mathbf{g}}^{w} = \sigma \left( {F_{w} \left( {{\mathbf{f}}^{w} } \right)} \right) $$

where $\sigma$ is the sigmoid activation function. To reduce overhead model complexity, here, we usually reduce number of f channel with appropriate reduction ratio *r*. Then we expand the outputs $g^{h}$ and $g^{w}$ as attention weights. Finally, the output of Coordinate Attention Block as the following Eq. (7):

7 $$ y_{c} (i,\;j) = x_{c} \;(i,j) \times g_{c}^{h}  (i) \times g_{c}^{w} (j) $$

#### Spatial attention

The pooling layer in the ordinary convolutional neural network directly uses max pooling or average pooling to compress the image information and reduce the amounts of operations to improve the accuracy rate. We need to further suppress the irrelevant regions in the input image and highlight the significant features of specific local areas, so we introduce the spatial attention module to further extract the key information.

**Figure 3:**
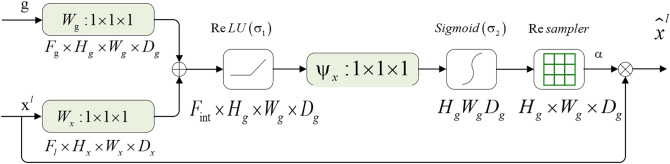
The attention module schematic. Input features (${\mathrm{x}}^{\mathrm{l}}$) are scaled with attention coefficients (α) computed in spatial attention. Spatial regions are selected by analyzing both activations and contextual information provided by the gating signal (g). ⊕ denotes add, and ⨂ denotes the elementwise multiplication.

To compute spatial attention, as in CBAM^34^, we apply the following Attention module schematic (shown as Fig. 3). We first apply average-pooling and max-pooling operations along the channel axis and concatenate them to generate two-feature maps g and *x*^*l*^ as inputs. The input g comes from decoder and *x*^*l*^ comes from the encoder.

First, two 1 × 1 × 1 kernel convolution layers are to generate two new feature maps ${g}_{i}\left({F}_{g}\times {H}_{g}\times {W}_{g}\times {D}_{g}\right)$ and ${x}_{i}^{l}\left({F}_{l}\times {H}_{x}\times {W}_{x}\times {D}_{x}\right)$, respectively, to capture edge information of tree-like structures in horizontal and vertical orientations. 1 × 1 × 1 convolutional operation on the feature map ${g}_{i}\left({F}_{g}\times {H}_{g}\times {W}_{g}\times {D}_{g}\right)$ in the downsampling layer from the N layer to get ${W}_{g}^{T}{g}_{i}$. Similarly, we get ${W}_{x}^{T}{x}_{i}^{l}$, we perform 1 × 1 × 1 convolution on the feature map ${x}_{i}^{l}\left({F}_{l}\times {H}_{x}\times {W}_{x}\times {D}_{x}\right)$ in the upsampling layer from N-1 layer. After that, we add feature maps ${W}_{g}^{T}{g}_{i}$ and ${W}_{x}^{T}{x}_{i}^{l}$ together and apply ReLU function to obtain $\sigma_{1} \left( {W_{x}^{T} x_{i}^{l} + W_{g}^{T} g_{i} + b_{g} } \right)$, which refers to $F_{{\text{int }}} \times H_{g} W_{g} D_{g}$ in the Fig. 3. Then, we apply another 1 × 1 × 1 convolutional computation to get $q_{att}^{l}$, the formulation is shown below:

8 $$ q_{att}^{l} = \psi^{T} \left( {\sigma_{1} \left( {W_{x}^{T} x_{i}^{l} + W_{g}^{T} g_{i} + b_{g} } \right)} \right) + b_{\psi } $$

where ${W}_{x}\in {\mathbb{R}}^{{F}_{l}\times {F}_{int}},{W}_{g}\in {\mathbb{R}}^{{F}_{g}\times {F}_{int}},\psi \in {\mathbb{R}}^{{F}_{int}\times 1}$ and bias terms ${b}_{\psi }\in {\mathbb{R}},{b}_{g}\in {\mathbb{R}}^{{F}_{int}}$ are computed using channel-wise 1 × 1 × 1 convolutions for the input tensors. Finally, using sigmoid activation function on $q_{att}^{l}$ to get attention weight $\alpha_{i}^{l}$, the formula is shown below:

9 $$ \alpha_{i}^{l} = \sigma_{2} \left( {q_{att}^{l} \left( {x_{i}^{l} ,\;g_{i} ;\;{\Theta }_{att} } \right)} \right) $$

This part is derivable and the value of attention coefficients can be adjusted by training. The formulation is shown below:

10 $$ \frac{{\partial \left( {\hat{x}_{i}^{l} } \right)}}{{\partial \left( {\Phi^{l - 1} } \right)}} = \frac{{\partial \left( {\alpha_{i}^{l} f\left( {x_{i}^{l - 1} ;\Phi^{l - 1} } \right)} \right)}}{{\partial \left( {\Phi^{l - 1} } \right)}} = \alpha_{i}^{l} \frac{{\partial \left( {f\left( {x_{i}^{l - 1} ;\Phi^{l - 1} } \right)} \right)}}{{\partial \left( {\Phi^{l - 1} } \right)}} + \frac{{\partial \left( {\alpha_{i}^{l} } \right)}}{{\partial \left( {\Phi^{l - 1} } \right)}}x_{i}^{l} $$

When attention coefficients, range 0–1, are multiplied with the feature map, values of irrelevant regions will become smaller (suppression), and the target regions will be larger (attention).

Our attention module infers attention mappings in two independents dimensional, channel and spatial orders, and then multiplies the attention mappings into input feature mappings for adaptive feature refinement. We then put attention module into CNN Architecture and gain the Dual Attention CNN Architecture.

**Figure 4:**
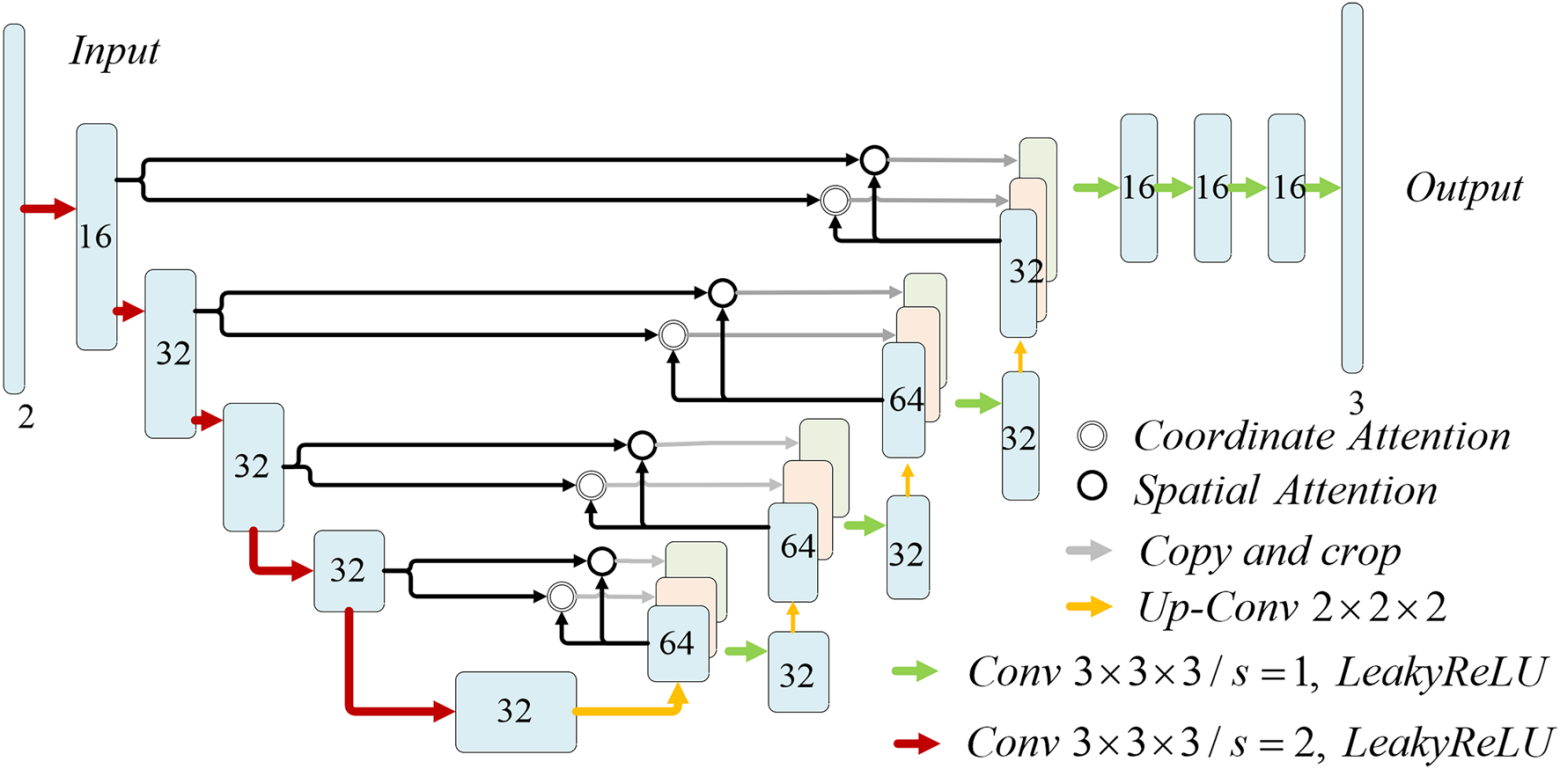
The dual attention CNN architecture. Each rectangle represented a 3D volume, generated from the previous volume by a 3D convolutional network layer. The spatial resolution of each volume was printed below. Fusion coordinate attention block and spatial attention block were added to the hop connection between each encoding stage and the corresponding decoding stage.

Dual Attention CNN Architecture is shown in Fig. 4, which splices the image pairs to be registered into 2-channel 3D image input. In the coding stage, 3 × 3 × 3 convolution with step stride 2 is used, and then the spatial dimension is reduced by Leaky ReLU activation. In the decoding phase, we alternate upsampling, 3 × 3 × 3 convolution (followed by Leaky ReLU activation), and join skip-connection. Fusion coordinate attention and spatial attention are added to the skip-connection between each encoding stage and the corresponding decoding stage. The feature maps of coordinate attention and spatial attention output are concatenated with the corresponding feature maps after up-sampling in the decoding stage. The high and low order features of spatial information are also collected. The spatial information from the encoding and corresponding decoding stages are fully extracted.

### DAVoxelMorph

**Figure 5:**
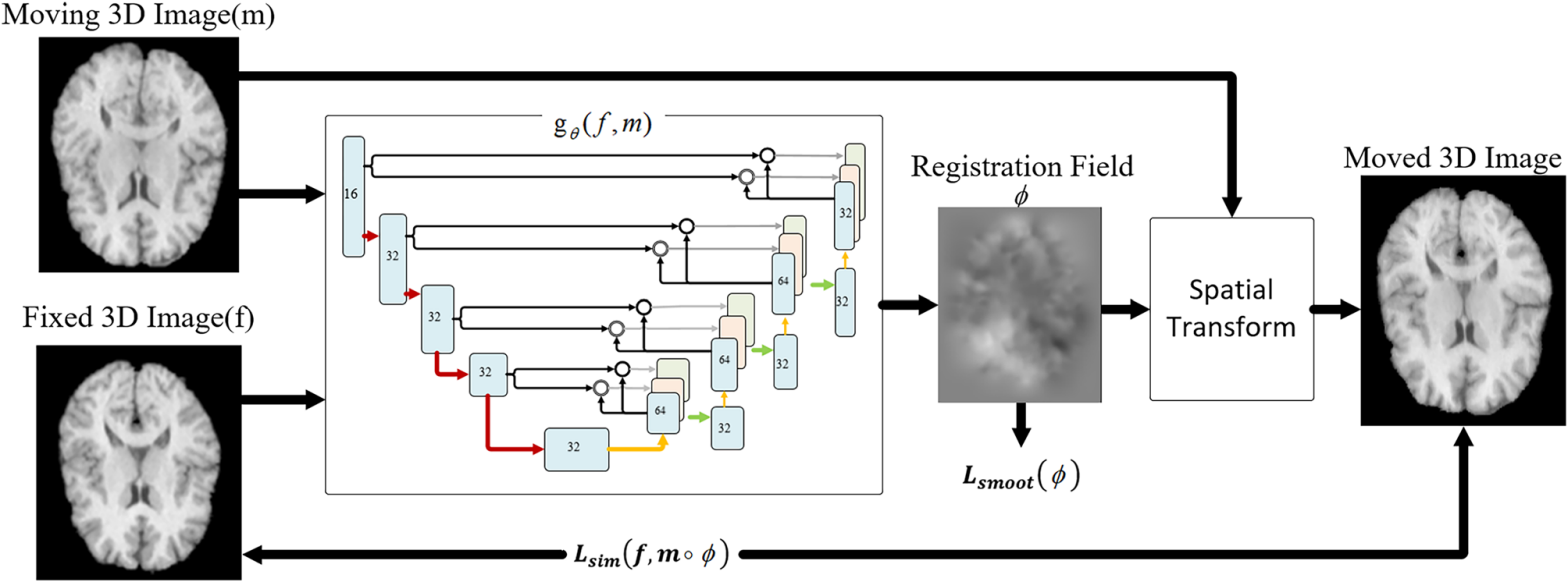
The overview of DAVoxelMorph model. We extract features and generate registration field through dual attention CNN architecture, and then register moving 3D image (M) to fixed 3D image (F) through spatial transform.

By adding the Dual Attention CNN Architecture into the standard VoxelMorph framework, the overview of DAVoxelMorph is demonstrated in the Fig. 5. To be specific, we propose to apply Dual Attention CNN Architecture to build registration field $\phi$ from the mapping of *f* to *m*, where *f*, *m* are two inputs of image volumes from n-dimensional space, *u* denotes the displacement field. We model a function gθ (f,m) = u using a convolutional neural network (CNN), where θ are network parameters. The registration field $\phi$ is stored in a n + 1-dimensional image. In other words, for each $p \in \Omega$, $u\left( p \right)$ is a displacement such that *f* (**p**) and [*m* ◦ *φ*](**p**) correspond to similar anatomical locations,

The network of p in the registration field so that voxel in m and f can correspond to similar anatomical locations. Similarly, $f\left( p \right)$ and $\left[ {m \circ \phi } \right]\left( p \right)$ denote the anatomic segmentation. Then, the network takes *f* and *m* as input, and computes *φ* using a set of parameters *θ*. We warp *m* to *m* ◦ *φ* using a spatial transformation function, enabling evaluation of the similarity of *m* ◦ *φ* and *f*.

### Loss functions

Our overall loss function $${\mathcal{L}}_{us}$$$$\left(\cdot ,\cdot ,\cdot \right)$$ uses input volumes and generated registration filed to evaluate the model. It consists of two components, including: (1) $${\mathcal{L}}_{sim}$$ to penalize differences of appearances; (2) $${\mathcal{L}}_{smooth}$$ to penalize local spatial variations in $$\phi$$:

11 $$ {\mathcal{L}}_{us} (f,m,\phi ) = {\mathcal{L}}_{sim} (f,m \circ \phi ) + {\mathcal{L}}_{smooth} $$

Let $\hat{f}({\mathbf{p}})$ and $[\hat{m} \circ \phi ]({\mathbf{p}})$ denote local mean intensity images: $\hat{f}({\mathbf{p}}) = \frac{1}{{n^{3} }}\sum\limits_{{{\mathbf{p}}_{i} }} f \left( {{\mathbf{p}}_{i} } \right)$, The local cross-correlation of $f$ and $m \circ \phi$ is written as:

12 $$ CC(f,m \circ \phi ) = \sum\limits_{{{\mathbf{p}} \in \Omega }} {\frac{{\left( {\sum\limits_{{{\mathbf{p}}_{i} }} {\left( {f\left( {{\mathbf{p}}_{i} } \right) - \hat{f}({\mathbf{p}})} \right)} \left( {[m \circ \phi ]\left( {{\mathbf{p}}_{i} } \right) - [\hat{m} \circ \phi ]({\mathbf{p}})} \right)} \right)^{2} }}{{\left( {\sum\limits_{{{\mathbf{p}}_{i} }} {\left( {f\left( {{\mathbf{p}}_{i} } \right) - \hat{f}({\mathbf{p}})} \right)^{2} } } \right)\left( {\sum\limits_{{{\mathbf{p}}_{i} }} {\left( {[m \circ \phi ]\left( {{\mathbf{p}}_{i} } \right) - [\hat{m} \circ \phi ]({\mathbf{p}})} \right)^{2} } } \right)}}} $$

A higher CC indicates a better alignment, yielding the loss function

13 $$ {\mathcal{L}}_{{\text{sim }}} (f,m,\phi ) = - CC(f,m \circ \phi ). $$

During the training of networks, previous studies used smoothness constraint of its spatial gradient^38,39^. However, we introduced a bending penalty term which regularizes the transformation, ${\mathcal{L}}_{smooth}$ = $\alpha P$, where P is the affine alignment, α = 0, and α is empirically determined to be 0.05 for all deformable image registration. Based on the general form of such a penalty term has been described by Wahba^40^, our bending penalty takes the following form:

14 $$ \begin{aligned}  {P = \frac{1}{V}\int_{0}^{X} {\int_{0}^{Y} {\int_{0}^{Z} {\left[ {\left( {\frac{{\partial^{2} {\mathbf{T}}}}{{\partial x^{2} }}} \right)^{2} + \left( {\frac{{\partial^{2} {\mathbf{T}}}}{{\partial y^{2} }}} \right)^{2} + \left( {\frac{{\partial^{2} {\mathbf{T}}}}{{\partial z^{2} }}} \right)^{2} } \right.} } } } \\ \hfill {\left. { \quad + 2\left( {\frac{{\partial^{2} {\mathbf{T}}}}{\partial xy}} \right)^{2} + 2\left( {\frac{{\partial^{2} {\mathbf{T}}}}{\partial xz}} \right)^{2} + 2\left( {\frac{{\partial^{2} {\mathbf{T}}}}{\partial yz}} \right)^{2} } \right]dxdydz} \\ \end{aligned} $$

## Experiments

### Datasets and preprocessing

We chose to experiment on publicly available data to demonstrate the performance of our approach. We evaluated our method and other registration methods on the brain MRI dataset LPBA40.LPBA40 datasets included a total of 56 structures labeled in MRI of 40 healthy, normal volunteers. Standard pre-processing steps were performed, including resampling all scans to a 256 × 256 × 256 grid with 1 mm isotropic voxels, affine special normalization, brain extraction using Freesurfer^41^, and crop the resulting images to 160 × 192 × 224.

### Evaluation metrics

We will evaluate our method and other registration methods from two perspectives. Registration mass and deformation field. For registration quality, we used an assessment based on volume overlap between organ segments and quantified it using the Dice score^14^. Comparing the mean and standard deviation of scores across the various registration methods, formulated as follows:

15 $$ {\text{Dice}} (A,B) = 2 \cdot \frac{|A \cap B|}{{|A| + |B|}} $$

where A is the reference image and B is the target image. A Dice score of 1 indicates that the structures are identical, and a score of 0 indicates that there is no overlap.

To evaluate the regularity of the registration field φ, the Jacobian matrix captures the local properties of φ around voxel p. We count all non-background voxels for which |Jφ(p)| ≤ 0, where the deformation is not diffeomorphic^42^. Formula is shown below:

16 $$ {\mathcal{N}}: = \Sigma \delta \left( {\det \left( {D\phi^{ - 1} } \right) < 0} \right) $$

### Implementation

We compare DAVoxelMorph with various registration methods that represent the most advanced registration performance at present. Two non-deep learning based methods include symmetric standardized SyN^37^ and NiftyReg^43^ in the publicly available advanced standardization tools (ANTs)^44^ software package. Two deep learning based methods, including VoxelMorph^12,38^ and CycleMorph^45^. There are four methods. Syn and NiftyReg are both non learning based methods. They achieve registration by optimizing the energy function of image pairs and have achieved remarkable success in various computational anatomy studies. VoxelMorph uses unsupervised learning, the loss performance reaches a high level in Dice coefficient, and the training time is greatly reduced. It can use auxiliary information and coarse label information to improve network performance. It is a very classic baseline network in the registration field. CycleMorph uses cyclic consistency to provide an implicit regularization to preserve the topology structure, which overcomes the problem that the registration method of deep learning usually cannot guarantee the preservation of topology.

For VoxelMorph implementation, we implemented our approach using PyTorch on a computer equipped with an Nvidia RTX A2000 GPU and an Intel Xeon Silver 4208 CPU. The Adam optimizer with a learning rate of 10^–4^, and a default of 50,000 iterations.

In our experiment, we split LPBA40 dataset into 30 training images and 10 testing images. We randomly choose one image from testing images as fixed image, and input 30 training images to the DAVoxelMorph model. We use Adam Optimizer with a learning rate of $4e^{ - 4}$, four scales with a default of 50,000 iterations, NCC as the image similarity loss function, and batch size as 1.

## Results and discussion

### Ablation study

**Figure 6:**
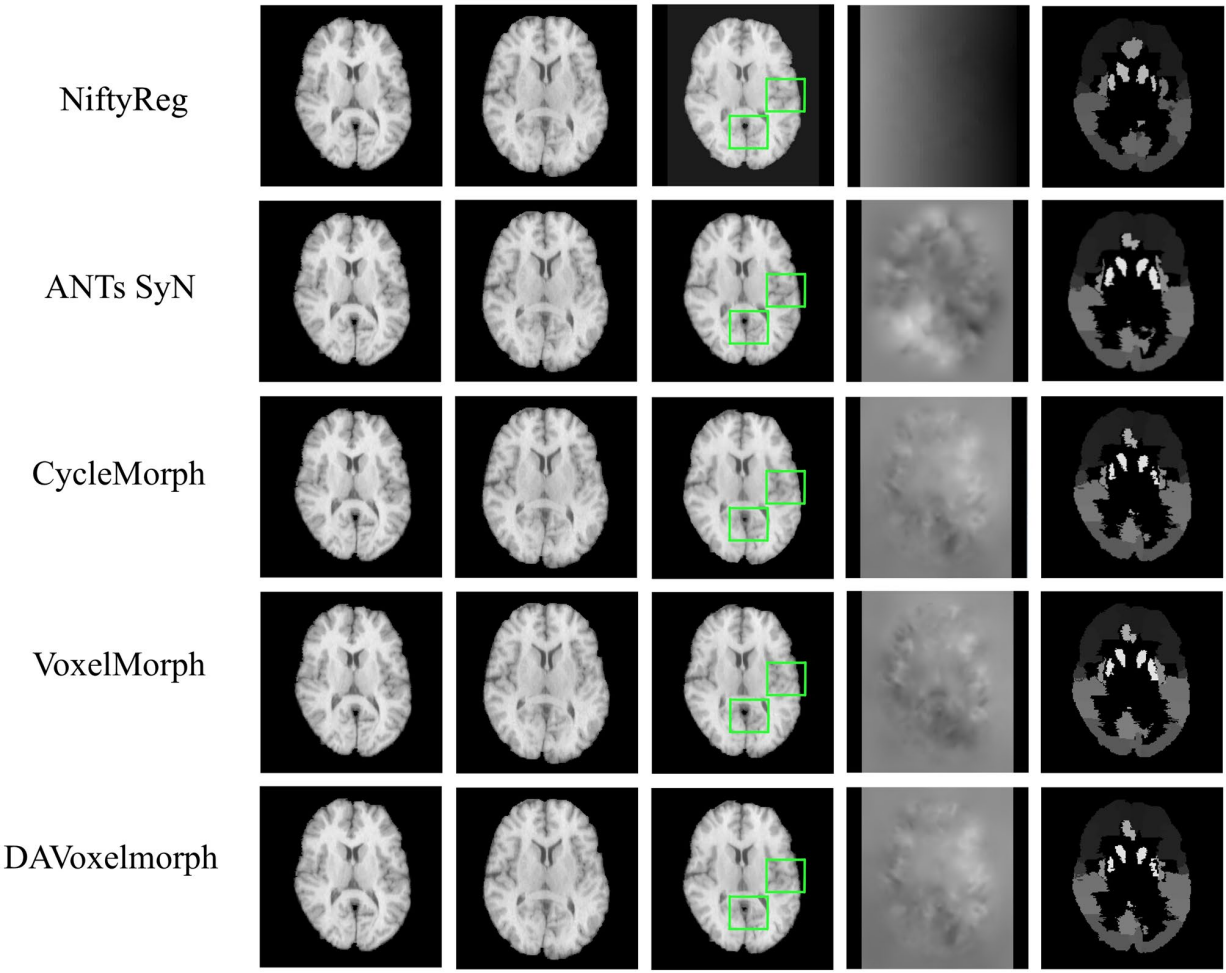
Registration results of different methods on LPBA40 dataset. The first column is the fixed image and the second column is the moving image. The third column shows the moved image after registration and in the area shown in the green box, DAVoxelMorph shows a better registration effect in detail. The fourth and the fifth column show the deformation field during the registration and label.

To demonstrate the performance of each key module in the DAVoxelMorph model, we perform a series of ablation experiments on the open dataset LPBA40^46^. The corresponding results of each module in DAVoxelMorph are demonstrated in the Table 1, and Fig. 6.

**Table 1:** Presents result on LPBA 40 dataset, including average Dice scores and percentage of locations with non-positive Jacobian. Standard deviations are presented in parentheses.

Method	Dice	% of ${\mathcal{N}}$
NiftyReg	0.694 (0.122)	0.549
VoxelMorph	0.703 (0.125)	0.355
ANTs SyN	0.707 (0.123)	0.137
CycleMorph	0.705 (0.133)	0.157
DAVoxelMorph (Bending Penalty only)	0.709 (0.122)	0.255
DAVoxelMorph (Dual Attention CNN Architecture only)	0.712 (0.126)	0.365
DAVoxelMorph (Dual Attention CNN Architecture and Bending Penalty)	0.714 (0.127)	0.345

Compared to DAVoxelMorph without bending penalty, DAVoxelMorph with bending penalty showed higher results of Dice score on LPBA 40 dataset, indicating the better performance.

Secondly, we compare the effectiveness of combination of Dual Attention CNN Architecture and bending penalty. Results of Dice score demonstrate that the combination model is superior to other models.

Taken together, bending penalty and Dual Attention CNN Architecture are two essential components in our DAVoxelMorph model, which makes the performance superior to other models.

### Comparison with other methods

The registration results on LPBA40 dataset with different quantitative evaluation measures are shown in Table 1. We compare our method with various methods.

Through experiments, on the open data set LBPA40 of brain MRI, the dice score and Jacobian matrix of the proposed method are higher than other methods. It is indicated that DAVoxelMorph proposed in this paper is best among all five methods in registration quality and three methods in deformation field (NiftyReg method does not generate deformation field). The configuration results and intermediate processes of the five methods in the experiment are shown in Fig. 6. DAVoxelMorph shows the best registration effect. The dice score and Jacobian matrix obtained by the five methods in the experiment are shown in Table 1. The Dice coefficient of this method is 0.714 (0.127), and the percentage of positions with non-positive Jacobian is 0.345, which is improved compared to the other methods.

## Conclusions

In our proposed method, the spatial attention mechanism calculates the attention map in the spatial dimension and focuses on the parts of the input image that need to be emphasized or suppressed. The coordinate attention mechanism embeds location information into channel attention to enable mobile networks to pay attention in a wider range. The dual attention CNN architecture enables our model to focus on the identification and positioning of tissues and organs that are more critical to the registration task. It also reduces or even filters the attention to other information, so to improve the efficiency and accuracy of registration. The bending penalty calculates the two-step degree of the deformation field. It further improves the registration quality by punishing the folding in the deformation field, promoting the affine transformation of the network locally, and enhancing the continuity and the global smoothness of the deformation field.

In conclusion, we propose a spatial attention enhanced, unsupervised learning method DAVoxelMorph for 3D deformable medical image registration. The results showed our model surpasses the basic VoxelMorph model, CycleMorph, as well as the ANTs SyN and NityReg in model sensitivity and registration accuracy with minimal computational overhead. The Dual Attention CNN Architecture in our model can continuously improve the registration performance under different datasets and training sizes while maintaining computational efficiency. Therefore, our proposed DAVoxelMorphmodel is a general learning model, but not limited to a particular image type or anatomy. It successfully speeds up the medical image analysis and processing pipelines, which can contribute to the clinical settings.

## Data Availability

The datasets generated during and/or analyzed during the current study are available in the https://www.loni.usc.edu/research/atlases.
